# Foam Properties and Detergent Abilities of the Saponins from *Camellia oleifera*

**DOI:** 10.3390/ijms11114417

**Published:** 2010-11-04

**Authors:** Yu-Fen Chen, Chao-Hsun Yang, Ming-Shiang Chang, Yong-Ping Ciou, Yu-Chun Huang

**Affiliations:** Department of Cosmetic Science, Providence University, 200, Chung-Chi Rd., Shalu, Taichung County, 43301, Taiwan; E-Mails: yfchen@pu.edu.tw (Y.-F.C.); chyang@pu.edu.tw (C.-H.Y.); sistericy@hotmail.com (M.-S.C.); g9824001@pu.edu.tw (Y.-P.C.)

**Keywords:** *Camellia oleifera*, saponin, foam, wetting, detergency

## Abstract

The defatted seed meal of *Camellia oleifera* has been used as a natural detergent and its extract is commercially utilized as a foam-stabilizing and emulsifying agent. The goal of this study was to investigate the foam properties and detergent ability of the saponins from the defatted seed meal of *C. oleifera*. The crude saponin content in the defatted seed meal of *C. oleifera* was 8.34 and the total saponins content in the crude saponins extract was 39.5% (w/w). The foaming power of the 0.5 crude saponins extract solution from defatted seed meal of *C. oleifera* was 37.1 of 0.5 SLS solution and 51.3% to that of 0.5% Tween 80 solution. The R5 value of 86.0% represents good foam stability of the crude saponins extracted from the defatted seed meal of the plant. With the reduction of water surface tension from 72 mN/m to 50.0 mN/m, the 0.5% crude saponins extract solution has wetting ability. The sebum-removal experiment indicated that the crude saponins extract has moderate detergency. The detergent abilities of the saponins from *C. oleifera* and *Sapindus mukorossi* were also compared.

## 1. Introduction

Saponins are a large family of structurally-related compounds of steroid or triterpenoid aglycone (sapogenin) linked to one or more oligosaccharide moieties by glycosidic linkage. The carbohydrate moiety consists of pentoses, hexoses, or uronic acids. The presence of both polar (sugar) and nonpolar (steroid or triterpene) groups provide saponins with strong surface-active properties [1]. Their physiochemical and biological properties feature structural diversity, which have led to a number of traditional and industrial applications [2–4]. Many saponins exhibit distinct foaming properties. They are also added to shampoos, liquid detergents, toothpastes and beverages as emulsifier and long-lasting foaming agent [5]. In addition, some pharmacological effects, such as molluscicidal [6], anti-inflammatory [7], antimicrobial [8], anthelmintic, antidermatophytic, antitussives and cytotoxic activities have been demonstrated in the saponins of plants [9].

*Camellia oleifera* is an important source of edible oil in tropical and sub-tropical regions of Asia, especially in China. It is used in cooking and as a medicine for the treatment of intestinal disorders and burn injuries [10–12]. The defatted seed meal, which contains saponins, is extensively used in aquaculture to eliminate unwanted fish and harmful insects in prawn ponds [13]. The major active ingredients of the defatted seed meal of *C. oleifera* are saponins. Previous phytochemical studies have identified various types of saponins, including camelliasaponin, theasaponin E1, theasaponin E2, and sasanquasaponin from the defatted seed meal of *C. oleifera* [11,14].

A recent trend in food and cosmetic preservation is to avoid the use of chemical agents, leaving scientists in search of natural antimicrobial alternatives. No study has yet characterized the foam properties and detergent abilities from the saponins of *C. oleifera*. Although foam generation has little to do with the cleansing ability of the detergent, it is an important criterion to evaluate detergent [15]. This study is thus aimed to investigate the foam properties and detergent abilities of the saponins from *C. oleifera*.

## 2. Results and Discussion

### 2.1. Saponins Extract from *C. oleifera*

The defatted seed meal powder of *C. oleifera* (15 g) was extracted with boiling water (450 mL) three times and the water extract was extracted successively with ethyl acetate and n-butanol. The percentage of crude saponins extract that was obtained from the defatted seed meal of *C. oleifera* was 8.34. The total saponins content in the crude saponins extract was 39.5% (w/w). Xi *et al*. [16] extracted the crude saponins from 11 commonly used saponin-rich traditional Chinese medicines (*Acanthopanax senticosus*, *Anemarrhena asphodeloides*, *Aralia taibaiensis*, *Asparrausi officinalis*, *Astragalus membranaceus*, *Ophiopogon japocicus*, *Panax ginseng*, *Panax notoginseng*, *Polygala tenuifolia*, *Polygonatum odoratum* and *Poria cocos*). The yields of crude saponins extract were all less than 6% (1.12%, 1.57%, 0.78%, 5.48%, 3.42%, 1.08%, 0.96%, 0.95%, 3.63%, 0.86% and 2.35% (w/w), respectively). The total saponins content were all less than 46%. These results show that the saponins content in the defatted seed meal of *C. oleifera* is higher than other traditional Chinese medicines.

### 2.2. Foam Power of the Crude Saponins

**Figure 1.**
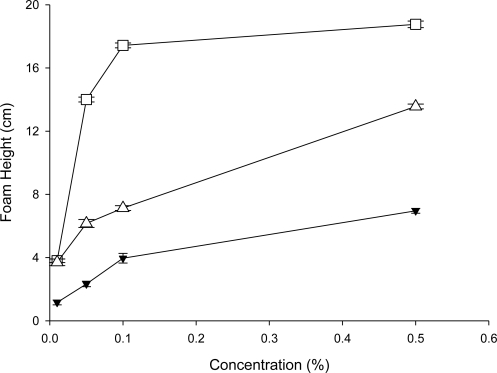
The foam power of various solutions. Data are expressed as mean ± SD of three independent experiments. (-▼-), Crude saponins extract; (-□-), SLS; (-▵-), Tween 80.

As shown in Figure 1, foam heights increased with increase in the aqueous solution concentrations. Among the three tested substances, sodium lauryl sulfate (SLS) ranked the highest for the foam height of the 0.5% solutions. The foaming height of the 0.5 crude saponins extract solution from *C. oleifera* was 37.1 of 0.5 SLS solution and was 51.3% with that of the 0.5% polyoxyethylene sorbate monooleate (Tween 80) solution. These results showed that 0.5% crude saponins extract solution from the defatted seed meal of *C. oleifera* possessed moderate foam power ability. Although foam generation has little to do with the cleansing ability of the detergents, it is extremely important for the user and is therefore an important criterion in evaluating detergents [15].

### 2.3. Foam Stability of the Crude Saponins

Foam produced by mechanical agitation is typically an unsteady thermodynamic system. When the foam set is resting, it will decay. The rate of weakening defines the stability of the foam [17]. The change of the foam height *versus* the time is shown in Table 1. The general aspect of the curves obtained shows little difference, which reflects a good foam stability *versus* time for 0.5% SLS, Tween 80 and the crude saponins extract solutions. Foam with R5 values higher than 50% can be regarded as metastable [18]. The R5 value of 0.5% saponins solution was 86.0%, representing the good foam stability of the saponins from the defatted seed meal of *C. oleifera*.

### 2.4. Wetting Ability of the Crude Saponins

**Figure 2.**
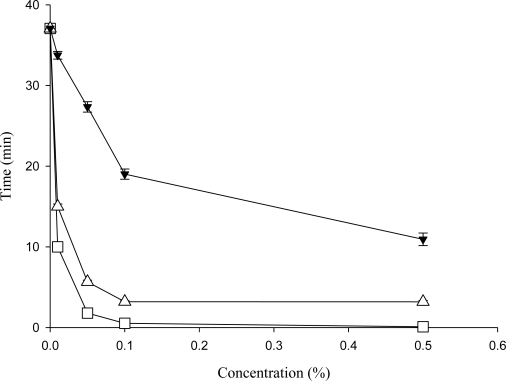
Variation of the wetting time *versus* the solution concentration. Data are expressed as mean ± SD of three independent experiments. (-▼-), Crude saponins extract; (-□-), SLS; (-▵-), Tween 80.

A detergent is something that increases the ability to displace air from a liquid or solid surface. The wetting phenomenon has aroused considerable commercial interest and plays a vital role in the removal of soil, dye, lubrication and printing by washing. The wetting ability of surface-active agent is commonly used to determine their comparative detergent efficacies. The Draves wetting data in Figure 2 shows that the wetting time decreased sharply with a solution concentration between 0.01–0.1%, followed by a slight drop after 0.1%. In terms of the wetting time of the 0.5% aqueous solution, the crude saponins extract solution needed 10.9 minutes to penetrate the cotton yarn while the SLS took just 0.1 minute. These data indicate that the wetting ability of the crude saponins extract of the plant was weaker than the general detergent, SLS.

### 2.5. Surface Tension of the Crude Saponins

**Figure 3.**
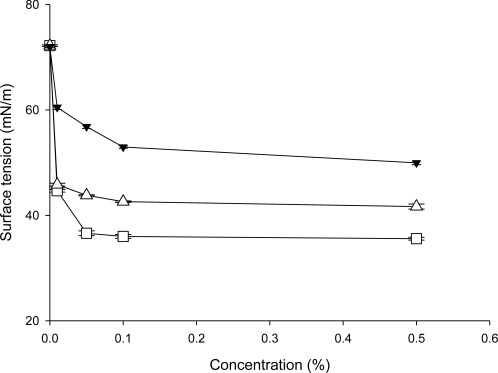
Variation of the surface tension *versus* the solution concentration. Data are expressed as mean ± SD of three independent experiments. (-▼-), Crude saponins extract; (-□-), SLS; (-▵-), Tween 80.

Wetting phenomena are complex and depend upon several processes and factors such as diffusion, surface tension, concentration and the nature of the surface being wet. Each wetting agent has to reduce surface tension. The reduction in the surface tension of water from 72 mN/m to 32–37 mN/m with the use of commercial shampoo is considered as a good detergent [15]. As shown in Figure 3, the surface tension of 0.5% aqueous solution dropped from 72.0 mN/m to 35.6 mN/m by SLS, to 41.7 mN/m by Tween 80 and to 50.0 mN/m by crude saponins extract, respectively.

### 2.6. Detergent Ability of the Crude Saponins

**Figure 4.**
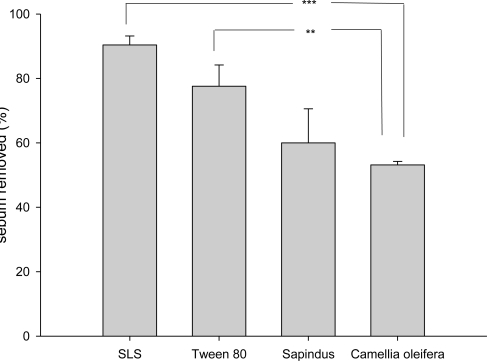
The percentage of sebum removed by detergents. Data are expressed as mean ± SD of three independent experiments. ** *P* < 0.01; *** *P* < 0.001. SLS: 0.5% sodium lauryl sulfate; Tween 80: 0.5% polyoxyethylene sorbate monooleate; Sapindus: 0.5% crude saponins extracted from *Sapindus mukorossi*; Camellia oleifera: 0.5% crude saponins extracted from *Camellia oleifera*.

The data indicated that crude saponins extract has the potential to be an effective detergent. However, for commercial application, a more direct examination of detergent ability should be made. The sebum removed from 0.5% SLS, Tween 80 and crude saponins extract solutions were 90.4%, 77.6% and 53.8%, respectively (Figure 4). These results indicate SLS has excellent detergency. Crude saponins extract and Tween 80 also show moderate detergency. According to the data from Yang *et al* [19], the crude saponin from *Sapindus mukorossi* also possessed moderate detergency (60%). It seems that the detergent ability of the saponin from *S. mukorossi* is superior to the saponin from *C. oleifera*. However, the saponins contents of them are different. The saponins content of the *S. mukorossi* crude saponins (85%) is double than the saponins content of the *C. oleifera* crude saponin (39.5%)

## 3. Experimental Section

### 3.1. Chemicals

The defatted seed meal of *C. oleifera* was purchased from Golden-Flower Tea Oil Company (Miaoli, Taiwan). This sample was originally collected from the mountains of central Taiwan in Miaoli County. The sample was milled to 100-mesh size in a Cyclone Mill (Tecator AB, Hoganas, Sweden). Quillaja saponin, inorganic salts and all other chemicals were from Sigma (St. Louis, MO, USA).

### 3.2. Determination of Total Saponins

The total saponins content of extract was determined by the vanillin-sulfuric acid method [20]. This extract was mixed with vanillin (8%, w/v) and sulfuric acid (72%, w/v). The mixture was incubated at 60 °C for 10 min, cooled in an ice water bath for another 15 min, followed by absorbance measurement at 538 nm. Quillaja saponin was used as a reference standard [21] and the content of total saponins was expressed as Quillaja saponin equivalents (QS μg/mg extract).

### 3.3. Foaming Properties

The method used for measuring foaming power and foam stability was developed by Ross and Miles [22]. A portion of the test solution was placed in a jacketed cylinder. Foam developed when a stream of a second portion of 200 mL test solution was added to the first portion of the test solution through a standard orifice from a 90 cm height. This resulted in turbulence and foam. The height of the foam generated was measured immediately and again after 5 min. The foam height at the initial stage indicates the foam power of the surfactant solution. The parameter R5, defined as the ratio of the height of the foam at 5 min to that at the initial stage, is proposed as an evaluation of foam stability [18].

### 3.4. Wetting Ability

The method used to measure wetting ability was developed by Draves [23]. In the test, a five gram skein of gray cotton yarn is submerged in the test solution. The time for the air in the yarn to be replaced by penetration of the solution is recorded. The end point is observed as the moment when the skein sinks. The unit of wetting ability is minute.

### 3.5. Surface Tension

Surface tension of the prepared surfactants was measured at room temperature (25 °C) using a Du Nouy tensionmeter (Sigma 703, KSV Instruments, USA), along with a 0.5% solution (w/v) in distilled water [24]. The surface tension of the added distilled water was 72.0 mN/m. The surfactants were aged for 30 min before further measurements.

### 3.6. Detergent Ability

The detergent ability was evaluated by a slightly modified version of the method developed by Thompson [15,25]. Hair tresses were obtained from a beauty salon. The tresses were prewashed with 5% SLS solution, dried and cut into 10 inch, 3 g swatches. The hair swatch (3 g) was suspended in 20 mL of 10% sebum solution (olive oil 20%, coconut oil 15%, stearic acid 15%, oleic acid 15%, paraffin wax 15% and cholesterol 20%) in hexane for 15 min with intermittent shaking. The swatch was removed, the solvent evaporated at room temperature and the dried hair swatch weighed to determine the sebum load. Each swatch was then split into two equal samples of 1.5 g each: one for the surfactant treatment and the other to act as an internal control to overcome the tress-to-tress variation in soil levels. The control swatch was left untreated. The test swatch was washed with 100 mL of the surfactant solution by the Finger Method described by Thompson *et al*. [25]. It was then dried using a hair dryer and further dried in an oven at 60 °C for 4 h to ensure uniform moisture content. The sebum remaining in the test swatch after surfactant treatment and that in the unwashed control one was then extracted using 20 mL of hexane in a stopper flask for 30 min on a rotary shaker. The hexane solution was then evaporated to dryness and the sebum extract from the test and control swatches was weighed. The detergent ability was evaluated as a percentage of sebum removed after surfactant treatment.

(1) $$\begin{array}{l} \text{Detergent ability}\;=\;100\;-\;(\text{T }\times \;100/\text{C})\\ \;\text{T}:\;\text{weight}\;\text{of}\;\text{sebum}\;\text{in}\;\text{test}\;\text{swatch}\;\\ \;\text{C}:\;\text{weight}\;\text{of}\;\text{sebum}\;\text{in}\;\text{control}\;\text{swatch}\; \end{array}$$

### 3.7. Statistical Analysis

All analytic measurements were taken at least in triplicate. Data are expressed as mean ± SD. Statistical comparison of means and simple correlation coefficients were conducted using the Student’s *t*-test in a general linear model (GLM) procedure of an SAS system (SAS Institute, Cary, NC).

## 4. Conclusions

From all these observation, it can be concluded that the saponins from *C. oleifera* show excellent foam properties and moderate detergency. These results are useful for the implementation of saponins from the *C. oleifera* in the detergent and cosmetic fields.

## Figures and Tables

**Table 1.** The foam heights of various solutions at the initial stage and after 5 min.

**Solution (0.5%)**	**Foam height (cm)**		R5*
	**0 min**	**5 min**	
SLS	18.8 ± 0.21	17.6 ± 0.19	93.6%
Crude saponins extract	4.57±0.10	3.93±0.05	86.0%
Tween 80	13.6 ± 0.15	13.1 ± 0.14	96.3%

* R5 is the ratio of the height of the foam at 5 min to that at 0 min.

Data are expressed as the mean ± SD of three independent experiments.

## References

[1] Makkar HPS, Siddhuraju P, Becker K (2007). Methods in Molecular Biology: Plant Secondary Metabolites.

[2] Price KR, Johnson IT, Fenwick GR (1987). The chemistry and biological significance of saponins in foods and feeding stuffs. CRC Crit. Rev. Food Sci. Nutr.

[3] Oakenfull D (1981). Saponins in food-A review. Food Chem.

[4] Martin RS, Briones R (1999). Industrial uses and sustainable supply of *Quillaja saponaria* (Rosaceae) saponins. Econ. Bot.

[5] Tanaka O, Tamura Y, Masuda H, Mizutani K, Waller GR, Yamasaki K (1996). Saponins Used in Food and Agriculture.

[6] Huang HC, Liao SC, Chang FR, Kuo YH, Wu YC (2003). Molluscicidal saponins from *Sapindus mukorossi*, inhibitory agents of golden apple snails, *Pomacea canaliculata*. J. Agric. Food Chem.

[7] Takagi K, Park EH, Kato H (1980). Anti-inflammatory activities of hederagenin and crude saponin isolated from *Sapindus mukorossi* Gaertn. Chem. Pharm. Bull.

[8] Tamura Y, Mizutani K, Ikeda T, Ohtani K, Kasai R, Yamasaki K, Tanaka O (2001). Antimicrobial activities of saponins of pericarps of *Sapindus mukurossi* on dermatophytes. Nat. Med.

[9] Sparg SG, Light ME, Staden J (2004). Biological activities and distribution of plant saponins. J. Ethnopharmacol.

[10] Huang Q, Shao L, He M, Chen H, Liu D, Luo Y, Dai Y (2005). Inhibitory effects of sasanquasaponin on over-expression of ICAM-1 and on enhancement of capillary permeability induced by burns in rats. Burns.

[11] Huang Q, He M, Chen H, Shao L, Liu D, Luo Y, Dai Y (2007). Protective effects of sasanquasaponin on injury of endothelial cells induced by anoxia and reoxygenation *in vitro.*. Basic Clin. Pharmacol. Toxicol.

[12] Chen H, He M, Huang Q, Liu D, Huang M (2007). Sasanquasaponin protects rat cardiomycytes against oxidatice stress induced by anoxia-reoxygenation injury. Eur. J. Pharmacol.

[13] Tang YA (1961). The use of saponin to control predaceous fish in shrimp ponds. Prog. Fish Cult.

[14] Chaicharoenpong C, Petsom A (2009). Quantitative thin layer chromatographic analysis of the saponins in tea seed meal. Phytochem. Anal.

[15] Mainkar AR, Jolly CI (2000). Evaluation of commercial herbal shampoos. Int. J. Cosmet. Sci.

[16] Xi MM, Hai CX, Tang HF, Chen MS, Fang KQ, Xin L (2008). Antioxidant and antiglycation properties of total saponins extracted from traditional Chinese medicine used to treat diabetes mellitus. Phytother. Res.

[17] Mousli R, Tazerouti A (2007). Direct method of preparation of dodecanesulfonamide derivatives and some surface properties. J. Surf. Deterg.

[18] Lunkenheimer K, Malysa K (2003). Simple and generally applicable method of determination and evaluation of foam properties. J. Surf. Deterg.

[19] Yang CH, Huang YC, Chen YF, Zhang MX (2010). Foam properties, detergent abilities and long-term preservative efficacy of the saponins from *Sapindus mukorossi.*. J. Food Drug Anal.

[20] Hiai S, Oura H, Nakajima T (1976). Color reaction of some sapogenins and saponins with vanillin and sulfuric acid. Planta Med.

[21] Shiau IL, Shih TL, Wang YN, Chen HT, Lan HF, Lin HC, Yang BY, Ko CH, Murase Y (2009). Quantification for saponin from a soapberry in cleaning products by a chromatographic and two colorimetric assays. J. Fac. Agr. Kyushu. Univ.

[22] Ross J, Miles GD (1941). An apparatus for comparison of foaming properties of soaps and detergents. J. Am. Oil Chem. Soc.

[23] Draves CZ (1939). Evaluation of wetting agents-official methods. Am. Dyestuff. Rep.

[24] Badawi AM, Mekawi MA, Mohamed AS, Mohamed MZ, Khowdairy MM (2007). Surface and biological activity of some novel cationic surfactants. J. Surf. Deterg.

[25] Thompson D, Lemaster C, Allen R, Whittam J (1985). Evaluation of relative shampoo detergency. J. Soc. Cosmet. Chem.

